# Iron‐Rich Food Consumption and Predictors Among Children Aged 6 to 23 Months in Ethiopia: A Frequentist and Bayesian Hierarchical Approach

**DOI:** 10.1002/fsn3.71651

**Published:** 2026-03-17

**Authors:** Girma Beressa, Kenenisa Beressa

**Affiliations:** ^1^ Department of Public Health Madda Walabu University Goba Ethiopia; ^2^ Department of English Addis Ababa University Addis Ababa Ethiopia

**Keywords:** children aged 6–23 months, Ethiopia, iron‐rich food, trends

## Abstract

Iron deficiency is a major public health issue in Ethiopia, especially in children aged 6 to 23 months. The 6 to 23 months period marks the introduction of complementary feeding alongside breast milk, when nutritional needs surge but intake often falls short, leading to high vulnerability. This study aimed to assess the weighted iron‐rich food consumption, trends, and predictors among children aged 6–23 months old in Ethiopia. This study used Ethiopian Demographic and Health Survey (EDHS‐2005–2019) data with a total weighted sample size of 7445 among children aged 6–23 months old. Bayesian multilevel logistic regression analysis using a Markov chain Monte Carlo simulation was conducted to identify predictors of good iron‐rich food consumption. The overall weighted proportion of iron‐rich food consumed among children aged 6–23 months in Ethiopia was 16.85% [95% CI: 15.01%, 18.86%]. In Ethiopia, children aged 6–23 months increased their intake of iron‐rich food from 10.29% [95% CI: 8.19%, 12.86%] in EDHS 2005 to 13.13% [95% CI: 11.11%, 15.47%] in EDHS 2011 and very sharply increased to 21.27% [95% CI: 18.54%, 24.29%] in EDHS 2016 and slightly increased to 23.77% [95% CI: 19.66%, 28.44%] in EDHS 2019. The findings indicated that being 12–23 month‐old children, having married parents, children born to mothers who completed primary education, secondary education, a higher education, being born to poorer family, middle wealth quintile, the richer family, richest family, and having antenatal care visits 1–3, ≥ 4 antenatal care visits were positively associated with iron‐rich food consumption. Nevertheless, children born to mothers ≥ 35 years old, residing in pastoral areas, and metropolitan areas were negatively significantly associated with good iron‐rich food intake among children. The weighted proportion of 16.85% indicates persistently low intake nationally, far below WHO minimum dietary diversity recommendations (≥ 50% for complementary feeding), signaling a high anemia risk in this vulnerable group. Significant associations (e.g., higher maternal education, wealthier quintiles, more ANC visits) underscore how enabling factors boost complementary feeding practices. Ethiopia should pursue strategies to boost iron‐rich food consumption.

## Graphical abstract

The overall weighted proportion of iron‐rich food intake was 16.85% [95% CI: 15.01%–18.86%]. Intake rose from 10.29% [8.19%–12.86%] (EDHS 2005) to 13.13% [11.11%–15.47%] (2011), sharply to 21.27% [18.54%–24.29%] (2016), and slightly to 23.77% [19.66%–28.44%] (2019). Being age 12–23 months, married parents, highly educated mothers, wealthier households, ≥ 4 ANC visits, mothers ≥ 35 years, and pastoral/metropolitan residence were significantly associated with iron‐rich food consumption.

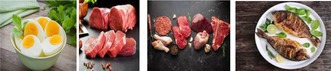

## 1 Introduction

Iron insufficiency is mostly caused by low intake, restricted absorption, and infection (Central Statistical Agency 2016). Iron deficiency anemia (IDA) is more common in children aged 6 to 23 months (Alemayehu et al. 2019). The previous study indicated that preschool children with inadequate iron consumption had slower growth, less immunity, and poorer cognitive development (Srivastava and Kumar 2021).

Existing evidence revealed that the proportion of iron‐rich food consumption in early childhood ranges from 21.41% in low‐ and middle‐income countries (LMICs) to 90% in high‐income countries (HICs) (De la Cruz‐Góngora et al. 2018; Tiruneh et al. 2020). Iron‐rich food intake among 6–23‐month‐old children was only 22% and 24% (Central Statistical Agency 2016; Indicators 2019). A nationwide study conducted in Ethiopia also indicated that iron‐rich food consumption among 6–23‐month‐old children was 21.41% and 27.14% (Terefe et al. 2024; Tiruneh et al. 2020).

The public health strategy supports initiatives to minimize child malnutrition in general, and child anemia in particular, by boosting dietary diversification, deworming, iron supplementation during pregnancy, and malaria control (Federal Democratic Republic of Ethiopia 2018). Individual‐level characteristics such as child age, maternal age, married, maternal education, wealth index, breastfeeding, and, as well as community‐level variables such as regions and community women's education, all revealed a strong association with iron consumption among Ethiopian children aged 6–23 months (Akalu et al. 2021; Eshetu et al. 2023; Terefe et al. 2024; Tiruneh et al. 2020).

The WHO promotes daily iron consumption as a suggested technique for the treatment and prevention of IDA (Thomas et al. 2020; Thompson et al. 2013). Although animal‐based meals are abundant in protein, fat, and minerals, they are not widely consumed in Ethiopia due to their expensive cost. As a result, low‐income nations, particularly Ethiopia, lack the physical and financial resources to purchase fortified animal products. A lack of animal‐based meals can lead to micronutrient deficiencies, including anemia (Abeshu et al. 2016).

The 6–23 month period marks the introduction of complementary feeding alongside breast milk, when nutritional needs surge but intake often falls short, leading to high vulnerability (Shagaro et al. 2021). A critical period of time for an infant's development and growth occurs before the age of two. This critical period may have an influence on how the children grow, learn, and develop in the future (Thrive 2011). This study's results can inspire activities and policies that accord with the UN's Sustainable Development Goals (SDGs) (WHO 2015). Moreover, these findings contribute to efforts to improve iron status and enhance nutritional and health outcomes for young children (Daru 2022; de Romana et al. 2021; Mensi and Udenigwe 2021). Identifying significant individual and community‐level predictors of iron‐rich food intake is crucial to improving iron‐rich consumption in Ethiopia. Therefore, this study aimed to assess the pooled iron‐rich food consumption, trends, and predictors among children aged 6–23 months old in Ethiopia using a novel approach, a Bayesian multilevel analysis.

## 2 Methods

### 2.1 Study Design, Data Source, and Participants

The data used for this study were accessed from the Ethiopian Demographic and Health Survey (EDHS) 2005–2019 data, which was used in a community‐based cross‐sectional study. This study employed data from the kids recode (KR) dataset, which had a weighted sample size of 7445 mother–child pairings. Ethiopia is divided into nine regional states (Afar, Amhara, Benishangul‐Gumuz, Gambella, Harari, Oromia, Somali, Southern Nations Nationalities and People's Region (SNNPR), and Tigray) and two city administrations. The emerging regions of Ethiopia were Afar, Somali, Benishangul‐Gumuz, and Gambella (Getinet et al. 2022). The standard EDHS data set has a large sample size, which helps to generate parameters (Croft et al. 2018). The source population was all children aged 6–23 months who lived with their mother, whereas the study population was all chosen or sampled living children aged 6–23 months who lived with their mother in the selected areas. To collect data from nine regional states and two municipal administrations, the EDHS employed a two‐stage sampling technique.

**FIGURE 1:**
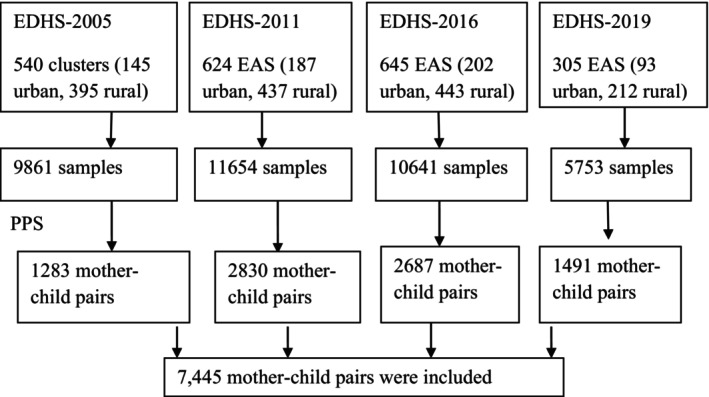
Schematic presentation of sampling procedure.

In the first phase, enumeration areas (EAs) were chosen with a probability proportionate to their size using the 2005–2019 primary health care (PHC) frameworks, with independent selection in each sample stratum. The second step is a systematic random sampling of residences within each cluster, or EA, followed by interviews with selected mother–child couples (Figure 1). Details have been described elsewhere (Central Statistical Agency 2005, 2011, 2016; “Federal Democratic Republic of Ethiopia, Mini Demographic and Health Survey,” 2019).

### 2.2 Data Collection Instruments

Child age, child sex, maternal age, marital status, religion, maternal education, wealth index, birth order, and antenatal care (ANC) were individual‐level factors, whereas residence and region were community‐level factors (Central Statistical Agency 2016). The wealth index was carried out using principal component analysis. Details have been described elsewhere (Beressa et al. 2024).

### 2.3 Outcome Assessment

If the children aged 6–23 months residing with their mother consumed at least one iron‐rich food at any time in the 24 h before the interview, among four food items, eggs, organ meat (liver, heart, or other organs), meat (beef, pork, lamb, or chicken), and fish were considered good consumption; otherwise, poor consumption (Croft et al. 2018; Terefe et al. 2024; Tiruneh et al. 2020).

### 2.4 Data Processing, Model Building, and Analysis

Stata 14 was used to conduct the data analysis. Descriptive statistics, such as frequency and percentage, were employed to describe study participants. The proportions and frequencies were weighted. All data analyses used the weighted using primary sampling unit, cluster (v021), sampling stratification, stratum (v023), and the individual sample weight (v005/1,000,000) to account for over‐ and under‐sampling. The DHS dataset is hierarchical, with children nested in households and households within clusters. A correlation matrix was used to assess multicollinearity among predictors. Variables with a bivariable multilevel mixed‐effects logistic regression analysis of less than 0.25 were included in the multivariable multilevel mixed‐effects logistic regression analysis to account for potential confounding effects (Austin and Tu 2004). Despite the *p*‐value assumption, a known factor such as region was included in the final model.

There were four models used: the null model (no predictors), model 1: individual‐level variables, model 2: community‐level factors, and model 3: individual and community‐level factors. Multilevel mixed‐effects logistic regression analyses were carried out to examine the relationship between explanatory variables and iron‐rich food consumption (yes = 1, no = 0). The data were analyzed using a complex sample survey multilevel mixed‐effects logistic regression analysis (melogit [pweight = swt] || v001). The Stata command “svyset” was used to establish survey data and estimate the percentage of iron‐rich food consumption. We evaluated the strength of the association between predictors and endpoint variables using the adjusted odds ratio (AOR), along with a 95% confidence interval.

A Markov chain Monte Carlo (MCMC) simulation was conducted using Bayesian multilevel mixed‐effects logistic regression analysis to identify predictors of good iron‐rich food consumption. Random‐walk Metropolis‐Hastings sampling was used. Details have been published elsewhere (Beressa et al. 2025). Adjusted odds ratio along with a 95% credible interval was used to estimate the strength of the association. The model's goodness‐of‐fit was evaluated using the Akaike Information Criterion (AIC), the Bayesian Information Criterion (BIC), and the log‐likelihood ratio (LLR). The model with the lowest AIC and BIC, as well as the highest LLR, was selected as the best fit.

The Median Odds Ratio (MOR), defined as the median value of the odds ratio between the areas at the lowest and highest risk when two clusters are randomly chosen, was used to quantify variation. MOR = e0.95√VA or exp. [√(2 × VA) × 0.6745], where VA represents the area‐level variation. The proportional change in variance (PCV) measures the variance in iron‐rich intake among children aged 6–23 months, which is explained by several variables. The PCV is calculated as Vnull‐VA/Vnull × 100. Where Vnull is the initial model's variance and VA is the model's variance with added terms. The intraclass correlation coefficient (ICC) estimates the variation in iron‐rich intake between clusters. It is computed as ICC = VA ÷ VA + 3.29 × 100%, where VA = area/cluster level variance (Merlo et al. 2006).

### 2.5 Ethical Approval and Consent to Participate

This complies with national guidelines, and ethical permission was received from Measure DHS using a data access request form. The EDHS data is available to the general public in various formats upon request from the Measure DHS website (www.measuredhs.com). Clinical trial number is not applicable. Ethics, consent to participate, and consent to publish declarations are not applicable. All methods were conducted in accordance with the relevant tenets of the Helsinki Declaration (WMA 2013).

## 3 Results

### 3.1 Sociodemographic and Economic Factors

This study employed a weighted sample of 7445 mother–child pairs from the EDHS 2005–2019 datasets (EDHS 2005 = 1283, EDHS 2011 = 2880, EDHS 2016 = 2224, and EDHS 2019 = 1058). Nearly two‐thirds EDHS 2005 = 855 (66.23%), EDHS 2011 = 1876 (64.40%), EDHS = 1905 (65.33%), and EDHS = 995 (66.47%) of children were in the categories of 12–23 month ages (Table 1).

**TABLE 1:** Sociodemographic and economic factors of study subjects, Ethiopia, EDHS 2005–2019 (*N* = 7445).

Variables	EDHS‐2005	EDHS‐2011	EDHS‐2016	EDHS‐2019	EDHS 2005–2019
	Frequency (%)	Frequency (%)	Frequency (%)	Frequency (%)	Frequency (%)
*Child's age (months)*					
6–11	436 (33.77)	1037 (35.60)	1011 (34.67)	502 (33.53)	2603 (34.96)
12–23	855 (66.23)	1876 (64.40)	1905 (65.33)	995 (66.47)	4842 (65.04)
*Child's sex*					
Male	634 (49.11)	1461 (50.15)	1440 (49.38)	763 (50.97)	3709 (49.82)
Female	657 (50.89)	1452 (49.85)	1476 (50.62)	734 (49.03)	3736 (50.18)
*Maternal age*					
15–19 years	91 (7.05)	193 (6.61)		107 (7.15)	483 (6.49)
20–34 years	908 (70.33)	2165 (74.17)		1152 (76.95)	5478 (73.58)
≥ 35 years	292 (22.62)	561 (19.22)		238 (15.90)	1484 (19.93)
*Marital status*					
Married	1202 (93.11)	2573 (88.33)	2738 (93.90)	1042 (93.65)	6838 (91.85)
Others^a^	89 (6.89)	340 (11.67)	178 (6.10)	95 (6.35)	607 (8.15)
*Religion*					
Orthodox	517 (40.05)	949 (32.58)	879 (30.14)	470 (31.40)	2541 (34.13)
Protestant	217 (16.81)	570 (19.57)	520 (17.83)	285 (19.04)	1330 (17.86)
Muslim	517 (40.05)	1313 (45.07)	1446 (49.59)	708 (47.29)	3428 (46.04)
Others^b^	40 (3.10)	81 (2.78)	71 (2.43)	34 (2.27)	146 (1.96)
*Maternal education*					
No education	969 (75.06)	1949 (66.91)	1743 (59.77)	731 (48.83)	4961 (66.64)
Primary education	217 (16.81)	788 (27.05)	806 (27.64)	524 (35.00)	1948 (26.17)
Secondary education	96 (7.44)	118 (4.05)	235 (8.06)	137 (9.15)	392 (5.27)
Higher education	9 (0.70)	58 (1.99)	132 (4.53)	105 (7.01)	144 (1.93)
*Wealth quintile*					
Poorest	346 (26.80)	862 (29.59)	987 (33.85)	455 (30.39)	2392 (32.13)
Poorer	242 (18.75)	531 (18.23)	486 (16.67)	245 (16.37)	1367 (18.36)
Middle	231 (17.89)	471 (16.17)	438 (15.02)	219 (14.63)	1277 (17.15)
Richer	207 (16.03)	460 (15.79)	358 (12.28)	201 (13.43)	1138 (15.29)
Richest	265 (20.53)	589 (20.22)	647 (22.19)	377 (25.18)	1271 (17.07)
*Birth order*					
1	229 (17.74)	530 (18.19)	618 (21.19)	331 (22.11)	1377 (18.50)
2–3	391 (30.29)	962 (33.02)	947 (32.48)	551 (36.81)	2405 (32.30)
≥ 4	671 (51.98)	1421 (48.78)	1351 (46.33)	615 (41.08)	3663 (49.20)
*ANC visits*					
No ANC visits	849 (68.03)	1551 (54.83)	890 (31.70)	364 (25.00)	3427 (47.50)
1–3	188 (15.06)	694 (24.53)	843 (30.02)	473 (32.49)	1882 (26.08)
≥ 4	211 (16.91)	584 (20.64)	1075 (38.28)	619 (42.51)	1906 (26.42)
*Residence*					
Urban	172 (13.41)	520 (17.85)	610 (20.92)	393 (26.25)	1190 (14.91)
Rural	1111 (86.59)	2393 (82.15)	2306 (79.08)	1104 (73.75)	6335 (85.09)
*Region*					
Agrarian	844 (65.78)	316 (10.85)	322 (11.04)	745 (49.77)	4513 (60.62)
Pastoral	335 (26.11)	232 (7.96)	260 (8.92)	544 (36.34)	2527 (33.94)
Metropolitan	104 (8.11)	328 (11.26)	268 (9.19)	208 (13.89)	405 (5.44)

Abbreviations: ANC, antenatal care; EDHS, Ethiopia demographic and health survey; SNNPR, Southern Nations, Nationalities, and Peoples' Region.

^a^ Others: never married, living together, widowed, divorced, not living, and together.

^b^ Others: catholic, traditional, and others.

### 3.2 Proportion of Iron‐Rich Food Consumption

The pooled proportion of iron‐rich food consumed among children aged 6–23 months in Ethiopia was 16.85% [95% CI: 15.01%, 18.86%]. In Ethiopia, children aged 6–23 months increased their intake of iron‐rich food from 10.29% [95% CI: 8.19%, 12.86%] in EDHS 2005 to 13.13% [95% CI: 11.11%, 15.47%] in EDHS 2011, very sharply increased to 21.27% [95% CI: 18.54%, 24.29%] in EDHS 2016, and slightly increased to 23.77% [95% CI: 19.66%, 28.44%] in EDHS 2019.

### 3.3 Individual and Community‐Level Predictors of Iron‐Rich Food Consumption Among Children Aged 6–23 Months Old

The null model has an intraclass correlation coefficient (ICC) of 8.77%. This suggested that children aged 6–23 months had varying iron‐rich intakes across clusters. The full model had the lowest AIC and highest LLR; hence, it was selected as the best‐fit model. The multivariable multilevel mixed‐effects logistic regression analyses indicated that being 12–23 month‐old children [AOR = 1.87, 95% CI: 1.45, 2.42], children born to mothers who completed primary education [AOR = 1.60, 95% CI: 1.25, 2.04], secondary education [AOR = 2.21, 95% CI: 1.50, 3.84], a higher education [AOR = 2.60, 95% CI: 1.32, 5.12], being born to the richer family [AOR = 1.61, 95% CI: 1.12, 2.29], richest family [AOR = 2.13, 95% CI: 1.32, 3.43], and having antenatal care visits greater than or equal to four [AOR = 1.40, 95% CI: 1.05, 1.87] were significantly positively associated with good iron‐rich food intake among children aged 6–23 months old. Nevertheless, children born to mothers aged 20–34 years old [AOR = 0.59, 95% CI: 0.37, 0.93] and mothers ≥ 35 years old [AOR = 0.52, 95% CI: 0.30, 0.92] were significantly negatively associated with good iron‐rich food consumption among children aged 6–23 months old (Table 2).

**TABLE 2:** Multivariable multilevel mixed‐effects logistic regression analyses predicting iron‐rich food consumption among children aged 6–23 months, Ethiopia, 2005–2019 (*N* = 7445).

Variable	Null model^a^	Model 1^b^	Model 2^c^	Model 3^d^
	AOR (95% CI)	AOR (95% CI)	AOR (95% CI)	AOR (95% CI)
*Child's age (months)*				
6–11		Ref		Ref
12–23		1.89 (1.46, 2.48)		1.87 (1.45, 2.42)**
*Maternal age*				
15–20 years		Ref		
20–34 years		0.62 (0.40, 0.98)		0.59 (0.37, 0.93)*
≥ 35 years		0.57 (0.32, 0.99)		0.52 (0.30, 0.91)*
*Marital status*				
Married		1.52 (1.03, 2.24)		1.45 (0.98, 2.15)
Others		Ref		Ref
*Maternal education*				
No education		Ref		Ref
Primary education		1.71 (1.34, 2.18)		1.60 (1.25, 2.04)**
Secondary education		2.57 (1.60, 4.11)		2.41 (1.50, 3.84)**
Higher education		2.96 (1.53, 5.73)		2.60 (1.33, 5.12)*
*Wealth quintile*				
Poorest		Ref		Ref
Poorer		1.42 (1.03, 1.96)		1.37 (0.99, 1.91)
Middle		1.39 (0.99, 1.98)		1.35 (0.94, 1.95)
Richer		1.63 (1.15, 2.31)		1.61 (1.12, 2.29)*
Richest		2.10 (1.44, 3.08)		2.13 (1.32, 3.43)*
*Birth order*				
1		Ref		Ref
2–3		0.04 (0.77, 1.40)		1.05 (0.78, 1.42)
≥ 4		0.96 (0.68, 1.35)		0.99 (0.70, 1.40)
*ANC visits*				
No ANC visits		Ref		Ref
1–3		1.14 (0.88, 1.47)		1.00 (0.78, 1.29)
≥ 4		1.69 (1.29, 2.22)		1.40 (1.05, 1.87)*
*Residence*				
Urban			Ref	Ref
Rural			0.35 (0.25, 0.50)	0.81 (0.49, 1.32)
*Region*				
Agrarian			1.19 (0.71, 2.01)	0.46 (0.85, 2.50)
Pastoral			0.44 (0.24, 0.78)	0.78 (0.42, 1.45)
Metropolitan			Ref	Ref
*Survey time*				
EDHS 2005			Ref	Ref
EDHS 2011			1.31 (0.88, 1.94)	**1.32 (0.89, 1.99)**
EDHS 2016			2.37 (1.61, 3.46)	2.13 (1.41, 3.20)**
EDHS 2019			2.45 (1.59, 3.80)	2.02 (1.28, 3.18)*
*Random effects*				
Variance (SE)	1.08	1.00	0.97	0.96
ICC%	8.77	6.96	8.41	6.79
PCV%	Ref	6.75	10.19	11.11
MOR	4.45	2.59	2.54	2.53
*Model fit statistics*				
AIC	7414.86	6927.95	7216.20	6876.22
BIC	7428.69	7044.95	7271.51	7034.53
Log likelihood (LL)	−3727.67	−3471.56	−3616.56	−3435.12
Deviance (−2 × LL)	7455.34	6943.12	7233.12	6870.24

*Note:* Statistical significant at *p < 0.05; **p < 0.001. Bold: statistically significant. Others: never married, living together, widowed, divorced, not living, and together.

Abbreviations: ANC, antenatal care; AOR, adjusted odds ratio; CI, confidence interval; EDHS, Ethiopia demographic and health survey; ICC, intraclass correlation coefficient; MOR, median odds ratio; PCV, proportional change in variance; Ref, reference; SE, standard error.

^a^ Without predictors.

^b^ Adjusted for individual‐level factors.

^c^ Adjusted for community‐level factors.

^d^ Full model.

### 3.4 Individual and Community‐Level Predictors of Iron‐Rich Food Consumption Among Children Aged 6–23 Months Old

The null model has an intraclass correlation coefficient (ICC) of 8.8%. This suggested that children aged 6–23 months had varying iron‐rich intakes across clusters. The full model had the lowest AIC and highest LLR; hence, it was selected as the best‐fit model. The Bayesian multivariable multilevel mixed‐effects logistic regression analyses indicated that being 12–23 month‐old children [AOR = 1.14, 95% CrI: 1.89, 2.39], having married parents [AOR = 1.26, 95% CrI: 1.08, 1.47], children born to mothers who completed primary education [AOR = 1.70, 95% CrI: 1.25, 1.85], secondary education [AOR = 2.90, 95% CrI: 1.64, 2.20], a higher education [AOR = 2.73, 95% CrI: 2.25, 3.39], being born to poorer family [AOR = 1.47, 95% CrI: 1.29, 1.66], middle wealth quintile [AOR = 1.54, 95% CrI: 1.43, 1.64], the richer family [AOR = 1.78, 95% CrI: 1.51, 2.09], richest family [AOR = 1.97, 95% CrI: 1.76, 2.23], and having antenatal care visits 1–3 [AOR = 1.14, 95% CrI: 1.02, 1.30], antenatal care visits greater than or equal to four [AOR = 1.65, 95% CrI: 1.41, 1.85] were significantly positively associated with good iron‐rich food intake among children aged 6–23 months old. Nevertheless, children born to mothers ≥ 35 years old [AOR = 0.84, 95% CrI: 0.70, 0.98], residing in pastoral areas [AOR = 0.86, 95% CrI: 0.74, 0.10], and residing in metropolitan areas [AOR = 0.72, 95% CrI: 0.56, 0.89] were significantly negatively associated with good iron‐rich food consumption among children aged 6–23 months old (Table 3).

**TABLE 3:** Multivariable Bayesian multilevel mixed‐effects logistic regression analyses predicting iron‐rich food consumption among children aged 6–23 months, Ethiopia, 2005–2019 (*N* = 7445).

Variable	Null model^a^	Model 1^b^	Model 2^c^	Model 3^d^
	AOR (95% CrI)	AOR (95% CrI)	AOR (95% CrI)	AOR (95% CrI)
*Child age (months)*				
6–11		Ref		Ref
12–23		2.07 (1.88, 2.29)		**1.14 (1.89, 2.39)**
*Maternal age (years)*				
15–19		Ref		
20–34		0.88 (0.79, 1.01)		0.90 (0.78, 1.04)
≥ 35		0.84 (0.67, 1.02)		**0.84 (0.70, 0.98)**
*Marital status*				
Married		1.36 (1.13, 1.61)		**1.26 (1.08, 1.47)**
Others		Ref		Ref
*Maternal education*				
No education		Ref		Ref
Primary education		1.81 (1.63, 1.98)		**1.70 (1.25, 1.85)**
Secondary education		1.85 (1.57, 2.20)		**2.90 (1.64, 2.20)**
Higher education		3.20 (2.49, 3.10)		**2.73 (2.25, 3.39)**
*Wealth quintile*				
Poorest		Ref		Ref
Poorer		1.52 (1.30, 1.77)		**1.47 (1.29, 1.66)**
Middle		1.61 (1.35, 1.88)		**1.54 (1.43, 1.64)**
Richer		1.62 (1.40, 1.92)		**1.78 (1.51, 2.09)**
Richest		1.93 (1.68, 2.27)		**1.97 (1.76, 2.23)**
*Birth order*				
1		Ref		Ref
2–3		0.97 (0.85, 1.09)		0.97 (0.89, 1.04)
≥ 4		0.98 (0.83, 1.18)		0.99 (0.70, 1.40)
*ANC visits*				
No ANC visits		Ref		Ref
1–3		1.30 (1.19, 1.42)		**1.14 (1.02, 1.30)**
≥ 4		2.03 (1.85, 2.22)		**1.65 (1.41, 1.85)**
*Residence*				
Urban			Ref	Ref
Rural			0.40 (0.33, 0.47)	**0.81 (0.70, 0.96)**
*Region*				
Agrarian			Ref	Ref
Pastoral			0.60 (0.71, 0.69)	**0.86 (0.74, 0.10)**
Metropolitan			0.87 (0.62, 1.20)	**0.72 (0.56, 0.89)**
*Survey time*				
EDHS 2005			Ref	Ref
EDHS 2011			1.30 (1.05, 1.59)	**1.21 (1.0, 1.44)**
EDHS 2016			1.85 (1.48, 2.31)	**1.50 (1.25, 1.73)**
EDHS 2019			2.03 (1.57, 2.60)	**1.46 (1.21, 1.71)**
*Random effects*				
Variance (SE)	0.31	0.25	0.30	0.24
ICC%	8.8	7.0	8.5	6.7
PCV%	Ref	19.58	4.07	26.31
MOR	1.71	1.61	1.69	1.58
*Model fit statistics*				
AIC	7414.86	6927.95	7216.20	6876.22
BIC	7428.69	7044.95	7271.51	7034.53
Log likelihood (LL)	−3727.67	−3471.56	−3616.56	−3435.12
Deviance (−2 × LL)	7455.34	6943.12	7233.12	6870.24

*Note:* Bold: statistically significant. Others: never married, living together, widowed, divorced, not living, and together.

Abbreviations: ANC, antenatal care; AOR, adjusted odds ratio; CrI, credible interval; EDHS, Ethiopia demographic and health survey; ICC, intraclass correlation coefficient; MCSE, Markov chain standard error; MOR, median odds ratio; PCV, proportional change in variance; Ref, reference.

^a^ Without predictors.

^b^ Adjusted for individual‐level factors.

^c^ Adjusted for community‐level factors.

^d^ Full model.

## 4 Discussion

This study aimed to assess the pooled iron‐rich food consumption, trends, and predictors among 6–23‐month‐old children in Ethiopia. The findings clearly pointed out that the pooled proportion of iron‐rich food consumption was 16.85% among children aged 6–23 months old. The findings revealed that being 12–23 month‐old children, children born to mothers who completed primary education, secondary education, a higher education, being born to the richer family, the richest family, and having antenatal care visits greater than or equal to four were significantly associated with good iron‐rich food consumption among children aged 6–23 months old.

The pooled proportion of good consumption of iron‐rich foods among children aged 6–23 months was 16.85%. This study was lower than studies conducted in Ethiopia (Terefe et al. 2024; Tiruneh et al. 2020), Rwanda (Eshetu et al. 2023), sub‐Saharan Africa (SSA) (Akalu et al. 2021), Sierra Leone (Semagn et al. 2023), and Afghanistan (Barekzai and Baraki 2021). This could be attributed to differences in media outlet exposure, socioeconomic status, and cultural status. Children aged 12–23 months old were 1.13 times more likely to consume good iron‐rich foods compared to their counterparts. This finding supported studies conducted in Ethiopia (Belay et al. 2022; Tiruneh et al. 2020) and SSA (Akalu et al. 2021). A study carried out in Ethiopia also found that as a youngster grows older, the likelihood of having anemic reduces (Gebremeskel et al. 2020).

Children born to mothers who completed a higher education level were 2.73 times more likely to consume iron‐rich meals compared to children born to mothers who did not have an education. This study supported research carried out in Ethiopia (Terefe et al. 2024; Tiruneh et al. 2020), Rwanda (Eshetu et al. 2023), Afghanistan (Barekzai and Baraki 2021), and India (Srivastava and Kumar 2021). This might be because educated women are exposed to a variety of media channels and realize the importance of diet for health. Moreover, educated women enhance their health‐seeking behaviors and obtain counseling services. Other relevant studies indicate that children born to illiterate women are more likely to develop anemia and consume fewer iron‐rich foods than children born to educated mothers, and vice versa (Eshetu et al. 2023; Gebreweld et al. 2019; Tiruneh et al. 2020).

Children born to mothers in the highest income quintile were 1.97 times more likely to consume iron‐rich meals than children born to the poorest mothers. This agreed with research undertaken in Ethiopia (Tiruneh et al. 2020), Rwanda (Eshetu et al. 2023), and SSA (Akalu et al. 2021). The possible explanation could be that children born to mothers from wealthy families have access to and can afford sources of iron‐rich food.

Children born to mothers who had antenatal care visits greater than or equal to four were significantly associated with good iron‐rich food consumption among children aged 6–23 months. To increase iron‐rich food intake among children, it is critical to improve ANC attendance and education during these visits. This includes specific dietary advice and help for mothers to include iron‐rich foods in their children's diets (Osei Bonsu et al. 2024; Semagn et al. 2023).

### 4.1 Policy Implications

The findings show crucial areas for policy intervention to improve iron‐rich dietary intake among Ethiopian young children. To address the information gaps surrounding iron‐rich food intake among Ethiopian children aged 6–23 months, future research should focus on many crucial areas: Research into the barriers that lower‐income families have in acquiring access to specific meals might lead to targeted responses (Belay et al. 2024; Beressa et al. 2024). Given the positive association between maternal education and children's consumption of iron‐rich foods, future study should look into the effectiveness of educational programs meant to increase mothers' nutritional knowledge. This might include community‐based initiatives that raise awareness of the need of iron in children's diets, as well as practical methods for including iron‐rich foods into meals (Beressa et al. 2024; Osei Bonsu et al. 2024). Integrating nutritional education into current health services, particularly during prenatal and postnatal care visits, helps encourage good eating habits.

### 4.2 The Strengths and Limitations of the Study

Because this dataset is a larger weighted national sample, it may be representative of the entire country. However, the data were collected via self‐reports, which may have contributed to recall and social desirability bias. There was a scarcity of literature on the study. Furthermore, because the study is cross‐sectional, a cause‐and‐effect link may not be established.

## 5 Conclusion

The finding revealed that there was a low consumption of iron‐rich foods among children aged 6–23 months in Ethiopia. These results highlight the importance of maternal education, household socioeconomic status, parental marital status, and antenatal care in improving iron‐rich food consumption, while also identifying specific demographic and geographic groups that may require targeted nutrition interventions. Ethiopia should pursue strategies to boost iron‐rich food consumption throughout these important phases of growth and development. Further research is required to attain robust findings.

## Author Contributions

G.B. participated in the conceptualization, formal analysis, methodology, software, validation, writing of the original draft, writing a review, and substantial editing. K.B. contributed to conceptualization, formal analysis, methodology, validation, writing of the original draft, writing a review, and substantial editing. The authors have read and approved the manuscript.

## Data Availability

The datasets used in this work are accessible upon reasonable request from the corresponding author.
